# Eligibility for heroin-assisted treatment (HAT) among people who inject opioids and are living with HIV in a Canadian setting

**DOI:** 10.1186/s13722-017-0104-y

**Published:** 2018-02-07

**Authors:** Jan Klimas, Huiru Dong, Nadia Fairbairn, Eugenia Socías, Rolando Barrios, Evan Wood, Thomas Kerr, Julio Montaner, M.-J. Milloy

**Affiliations:** 1 British Columbia Centres on Substance Use and Excellence in HIV/AIDS, St. Paul’s Hospital, 608-1081 Burrard Street, Vancouver, BC V6Z 1Y6 Canada; 2 Department of Medicine, University of British Columbia, St. Paul’s Hospital, 608-1081 Burrard Street, Vancouver, BC V6Z 1Y6 Canada; 3 School of Medicine, University College Dublin, Health Sciences Centre, Belfield, Dublin 4, Ireland

**Keywords:** Substance-related disorders, Heroin, HIV/AIDS, Illicit drug use, Opioid agonist treatment

## Abstract

**Objectives:**

A growing body of evidence supports the effectiveness of injectable diacetylmorphine (i.e., heroin) for individuals with treatment-refractory opioid use disorder. Despite this evidence, and the increasing toll of opioid-associated morbidity and mortality, it remains controversial in some settings. To investigate the possible contribution of heroin-assisted treatment (HAT) to HIV treatment-related outcomes, we sought to estimate the proportion and characteristics of HIV-positive people who inject opioids that might be eligible for HAT in Vancouver, Canada.

**Methods:**

We used data from a prospective cohort of people living with HIV who use illicit drugs in Vancouver, Canada. Using generalized estimating equations (GEE), we assessed the longitudinal relationships between eligibility for HAT, using criteria from previous clinical trials and guidelines, with behavioural, social, and clinical characteristics.

**Results:**

Between 2005 and 2014, 478 participants were included in these analyses, contributing 1927 person-years of observation. Of those, 94 (19.7%) met eligibility for HAT at least once during the study period. In a multivariable GEE model, after adjusting for clinical characteristics, being eligible for HAT was positively associated with homelessness, female gender, high-intensity illicit drug use, drug dealing and higher CD4 count.

**Conclusions:**

In our study of HIV-positive people with a history of injection drug use, approximately 20% of participants were eligible for HAT at ≥ 1 follow-up period. Eligibility was linked to risk factors for sub-optimal HIV/AIDS treatment outcomes, such as homelessness and involvement in the local illicit drug trade, suggesting that scaling-up access to HAT might contribute to achieving optimal HIV treatment in this setting.

## Background

A growing body of evidence, including findings from randomised controlled trials (RCT) in Europe and North America, and systematic reviews by the Cochrane Collaboration and others, supports the effectiveness of injectable diacetylmorphine (i.e., prescribed heroin) for treatment-refractory opioid use disorder [1–3]. Despite this support, heroin-assisted treatment (HAT) remains controversial and unavailable in most settings [4]. However, given the growing amount of evidence and the increasing toll of opioid-associated morbidity and mortality, efforts to develop guidance on injectable opioid agonist treatments for opioid use disorder, including medications that do not face the same regulatory barriers as diacetylmorphine (e.g., hydromorphone), are under way in some settings [5].

Medical management of people living with HIV (PLHIV), with co-occurring substance use disorder (SUD), poses many challenges. There remains low coverage of medically-proven treatment for SUD in many settings [6, 7]; furthermore, HIV-positive people who use illicit drugs (PWID) have not fully benefitted from scale-up of HIV testing and antiretroviral treatment (ART), a strategy commonly referred to treatment-as-prevention (TasP), has resulted in reduced rates of HIV-associated morbidity, mortality and viral transmission [8]. As a result of sub-optimal HIV treatment outcomes and barriers to accessing harm reduction supplies, such as sterile syringes, HIV outbreaks among PWID are common and ongoing in many settings [9, 10].

Despite the demonstrated benefits of oral opioid agonist therapy (OAT, i.e. methadone, buprenorphine/naloxone) on reducing illicit opioid use and HIV risk behaviours and promoting optimal HIV treatment outcomes [11, 12], gaps in SUD treatment persist, especially for PWID living with HIV [6, 13]. The limitations of oral OAT are many and significant, specifically the attrition that has been shown to increase the mortality risk during the induction phase onto methadone treatment, during the first year and during the time immediately after leaving treatment.[ENTER REF SORDO] In this respect, several RCTs (e.g., NAOMI, SALOME) have demonstrated the potential benefits of HAT for people with treatment-refractory opioid use disorder, including decreased levels of used syringe sharing, reduced illicit drug use, criminal activity and increased engagement with healthcare [1–3, 14]. However, we are unaware of any study to explore the potential uptake of HAT in the HIV-positive population. Thus, we sought to estimate the prevalence and characteristics of HIV-positive individuals that might be eligible for HAT in Vancouver, Canada.

## Methods

Data for these analyses were derived from a prospective cohort of people who use illicit drugs and live with HIV in Vancouver, Canada, the AIDS Care Cohort to evaluate Exposure to Survival Services (ACCESS), which has been described elsewhere [15–17]. In brief, ACCESS is a cohort of HIV-seropositive adults who have used at least one illicit drug (other than or in addition to cannabis) in the month prior to recruitment. Individuals are recruited from community settings via snowball sampling and outreach techniques. At baseline and each biannual follow-up interview, participants complete interviewer-administered questionnaires that assess socio-demographic, drug use and other related behaviours, characteristics, or exposures. This includes a nursing examination and phlebotomy for HIV clinical monitoring. In addition to the interview data, we also accessed linked (using a government-issued identifier, i.e., Personal Health Number—PHN) data from a comprehensive retrospective and prospective HIV clinical monitoring profile, including all plasma HIV-1 RNA viral loads, CD4 cell counts and records of all antiretroviral therapy (ART) dispensations, available from the Drug Treatment Program at the BC Centre for Excellence in HIV/AIDS (BC-CfE), as described previously [18]. The BC-CfE is responsible for dispensing ART, provided at no charge, to all people living with HIV in the province. The records include all clinical measures conducted through the study or by a participant’s physician or healthcare provider.

Participants are compensated at each study interview with a $30 CDN stipend. The University of British Columbia/Providence Healthcare Research Ethics Board approved the study and all participants provided written informed consent at baseline.

We included all participants who completed at least one study visit between December 1, 2005 and May 31, 2014 and who were older than 18 years old and reported ever injecting drugs at least once at the baseline interview. Our primary outcome of interest was being eligible for HAT as per the historical criteria from previous published RCTs [1–3], that defined HAT eligibility variably as: (a) currently residing in the study area (i.e., the city of Vancouver); (b) current regular injection of illicit opioids (i.e., ≥ one time in the previous 6 months); (c) at least two self-reported prior SUD treatment attempts, including one episode of OAT (i.e., methadone or buprenorphine/naloxone); (d) at least 5 years of illicit opioid use; and (e) poor health, or psychosocial functioning, defined as a self-reported mental health diagnosis. At each interview period of the present study, we determined if an individual was eligible for HAT during that period by evaluating each criterion using responses given to relevant interview questions, specifically: (a) current residence in the City of Vancouver; (b) ≥ weekly injection of an opioid (e.g., heroin, methadone, prescription opioids, etc.) in the last 6 months (yes vs. no); (c) number of times during the study period reporting being engaged in any SUD treatment and any OAT included if (1) positive response to at least two of the options, or at least two positive responses for “number of times in treatment” at baseline, including one MMT programme, *or* included if (2) participants reported at least two prior “attempts at treatment” over follow-up, defined as not being currently in treatment but treatment in the last 6 months (≥ 2 vs. < 2); (d) number of years since initiation of illicit opioid use (≥ 5 vs. < 5 years); and (e) reporting a mental health diagnosis (Have you been diagnosed with a mental health issue in the last 6 months? Yes vs. no), or baseline depression, as measured by the Center for Epidemiologic Studies Depression scale (CES-D) (score of ≥ 16 vs. score of < 16). Participants had to fulfill all the above criteria to be deemed eligible for HAT.

We also defined a number of explanatory variables, including: age (per year older); gender (male vs. non-male); Caucasian ethnicity/ancestry (yes vs. no); hepatitis C virus antibody status (positive vs. negative); number of years of using injection heroin at baseline; homelessness (yes vs. no); relationship status (legally married/common law/regular partner vs. other); highest level of education completed (≥ high school diploma vs. < high school diploma); formal employment (yes vs. no, i.e., regular job, temporary job, or self-employed); money spent on drugs per day (≥ $50 per day vs. < $50 per day); drug dealing (yes vs. no); ≥ daily non-injection cocaine use (yes vs. no); ≥ daily non-injection heroin use (yes vs. no); ≥ daily crack use (yes vs. no); ≥ daily methamphetamine use (yes vs. no); non-fatal overdose (yes vs. no); lent used syringe (yes vs. no); recent incarceration (yes vs. no); engagement in any form of unprotected sex (yes vs. no); exchange of sex for gifts, food, shelter, clothes, etc. (yes vs. no); being a victim of violence, defined as having been attacked or assaulted (yes vs. no) [19–21]. All time-updated variables refer to behaviours or exposures in the 6-month period prior to the study interview.

We also included the following data based on the confidential linkage (using PHN identifier) to the local HIV clinical monitoring registry and ART dispensary: HIV-1 RNA plasma viral load (VL), using the median of all observations in the previous 6 months or, if none, the most recent observation, dichotomised at > 50 versus ≤ 50 copies/mL; ART engagement, using the number of days of ART dispensed in the previous 6 months (dichotomized at ≥ 1 vs. 0 day); and CD4 cell count, using the median of all observations in the previous 6 months or, if none, the most recent observation (expressed per 100 cells/mL) [22].

As a first step, we described the study sample at baseline stratified by eligibility for HAT. We used Chi square test and Fisher’s exact test to compare categorical variables and Wilcoxon’s rank-sum test to compare continuous variables.

To evaluate the association between eligibility for HAT and each of the explanatory variables of interest, we built a statistical model, using generalised estimating equation (GEE), assuming a binomial distribution, a logit-link function and an exchangeable working correlation structure. We first build bivariable GEE to examine the association between being eligible for HAT and each of the explanatory variables. To fit the final multivariable model, we applied an a priori-defined backward model selection approach based on examination of quasilikelihood under the independence model criterion statistic (QIC). We first included all explanatory variables that were associated with the outcome at the level of *p* < 0.10 in bivariable analyses in a full model. From the QIC of the model, we excluded the variable with the largest *p* value and constructed a reduced model. We proceeded this iterative method and chose the multivariable model with the lowest QIC value [23]. All *p* values were two-sided. All statistical analyses were performed using the SAS software version 9.4 (SAS, Cary, NC, USA).

## Results

Between December 2005 and May 2014, 852 individuals were recruited into the cohort, of whom 478 (56.1%) participants satisfied all criteria, including age and injection drug use, and were included in the analysis. They contributed 3495 observations, or a median of 7 interviews [inter-quartile range (IQR): 3–11] during follow-up. Of those, 94 (19.7%) were deemed to be eligible for HAT at least once through study period: 32 reported eligible for once, 19 twice, 11 thrice, and 32 more than thrice. Baseline characteristics are reported in Table 1. As shown, most participants included in this analysis were Caucasian (273, 57.1%), males (321, 67.2%), with median age of 43.1 (IQR 36.7–47.9) years.

**Table 1:** Baseline characteristics of participants who inject opioids and live with HIV in Vancouver, BC, stratified by the baseline HAT eligibility status

Characteristic	Total (%) (*n* = 478)	HAT eligibility		*p* value
		Yes (*%*) 45 (9.4)	No (%) 433 (90.6)	
Age (median, inter-quartile range-IQR)^b^	43.1 (36.7–47.9)	36.7 (34.2–43.7)	43.5 (37.9–48.1)	< 0.001
Gender				
Male	321 (67.2)	23 (51.1)	298 (68.8)	0.016
Non-male	157 (32.8)	22 (48.9)	135 (31.2)	
Ethnicity				
Caucasian	273 (57.1)	29 (64.4)	244 (56.4)	0.296
Non-Caucasian	205 (42.9)	16 (35.6)	189 (43.6)	
Homelessness^a^				
Yes	169 (35.4)	22 (48.9)	147 (33.9)	0.026
No	305 (63.8)	21 (46.7)	284 (65.6)	
Relationship status^a^				
Legally married/common law/regular partner	126 (26.4)	15 (33.3)	111 (25.6)	0.228
Other	339 (70.9)	28 (62.2)	311 (71.8)	
Highest level of education completed				
≥ high school diploma	229 (47.9)	23 (51.1)	206 (47.6)	0.512
< high school diploma	241 (50.4)	20 (44.4)	221 (51.0)	
Employment^a,c^				
Yes	101 (21.1)	4 (8.9)	97 (22.4)	0.035
No	377 (78.9)	41 (91.1)	336 (77.6)	
Money spent on drugs p/day^c^				
≥ $50 p/day	315 (65.9)	39 (86.7)	276 (63.7)	< 0.001
< $50 p/day	158 (33.1)	5 (11.1)	153 (35.3)	
Drug dealing^a^				
Yes	148 (31.0)	25 (55.6)	123 (28.4)	< 0.001
No	330 (69.0)	20 (44.4)	310 (71.6)	
Years of using heroin injection heroin at baseline (median, IQR)^b^				
Number of years	14.8 (7.3–22.6)	15.7 (8.7–20.4)	14.4 (7.3–22.7)	0.640
Daily non-injection cocaine use^a,c^				
Yes	2 (0.4)	0 (0.0)	2 (0.5)	1.000
No	476 (99.6)	45 (100.0)	431 (99.5)	
Daily non-injection heroin use^a,c^				
Yes	4 (0.8)	1 (2.2)	3 (0.7)	0.328
No	474 (99.2)	44 (97.8)	430 (99.3)	
Daily crack use^a^				
Yes	181 (37.9)	25 (55.6)	156 (36.0)	0.010
No	297 (62.1)	20 (44.4)	277 (64.0)	
Daily crystal meth use^a^				
Yes	19 (4.0)	5 (11.1)	14 (3.2)	0.025
No	459 (96.0)	40 (88.9)	419 (96.8)	
Overdose^a^				
Yes	32 (6.7)	7 (15.6)	25 (5.8)	0.012
No	446 (93.3)	38 (84.4)	408 (94.2)	
Lent syringe^a,c^				
Yes	17 (3.6)	3 (6.7)	14 (3.2)	0.210
No	460 (96.2)	42 (93.3)	418 (96.5)	
Recent incarceration^a^				
Yes	75 (15.7)	12 (26.7)	63 (14.5)	0.034
No	402 (84.1)	33 (73.3)	369 (85.2)	
Engaged in any form of unprotected sex^a,c^				
Yes	46 (9.6)	5 (11.1)	41 (9.5)	0.790
No	429 (89.7)	40 (88.9)	389 (89.8)	
Exchanged sex for gifts, food, shelter, clothes, etc.^a^				
Yes	72 (15.1)	15 (33.3)	57 (13.2)	< 0.001
No	403 (84.3)	30 (66.7)	373 (86.1)	
Attacked, assaulted, or suffered violence^a^				
Yes	98 (20.5)	12 (26.7)	86 (19.9)	0.293
No	377 (78.9)	33 (73.3)	344 (79.4)	
HCV^c^				
Yes	429 (89.7)	40 (88.9)	389 (89.8)	0.797
No	49 (10.3)	5 (11.1)	44 (10.2)	
Plasma HIV-1 RNA viral load > 50 c/mL				
Yes	313 (65.5)	31 (68.9)	282 (65.1)	0.656
No	162 (33.9)	14 (31.1)	148 (34.2)	
On ART (≥ 1 day)^a^				
Yes	283 (59.2)	28 (62.2)	255 (58.9)	0.665
No	195 (40.8)	17 (37.8)	178 (41.1)	
CD4+ cell count per 100 cells^a,b^				
Median, IQR	3.2 (2.0–4.8)	3.2 (1.6–5.0)	3.2 (2.0–4.8)	0.696

^a^All behavioural variables refer to the 6 months prior to the follow-up questionnaire

^b^Continuous variable, *p* value is generated from Wilcoxon rank-sum test

^c^*p* value is generated from Fisher’s exact test because of small cell count

As shown in Table 2, the following variables were positively associated with being eligible for HAT: homelessness [adjusted odds ratio (AOR) 1.47; 95% confidence interval (CI) 1.07–2.01]; drug dealing in the last 6 months (AOR 1.76; 95% CI 1.33–2.31); ≥ daily non-injection heroin use (AOR 8.18; 95% CI 1.25–53.56); ≥ daily crack use (AOR1.35; 95% CI 1.01–1.82); and CD4 cell count (per 100 cells/mL increase, AOR 1.08; 95% CI 1.00–1.17). Male gender was negatively associated with the outcome (AOR 0.50; 95% CI 0.31–0.82).

**Table 2:** Bivariable and multivariable GEE analysis of factors associated with HAT eligibility among people who live with HIV

Characteristic	Bivariable		Multivariable	
	Odds ratio (95% CI)	*p* value	Odds ratio (95% CI)	*p* value
Age				
(Per 10 years older)	0.97 (0.94–1.01)	0.101		
Gender				
(Male vs. female/non-male)	0.49 (0.31–0.80)	0.004	0.50 (0.31–0.82)	0.006
Ethnicity				
(Caucasian vs. other)	1.39 (0.84–2.28)	0.198		
Homelessness^a^				
(Yes vs. no)	1.61 (1.21–2.16)	0.001	1.47 (1.07–2.01)	0.016
Relationship status^a^				
(Legally married/common law/regular partner vs. other)	0.82 (0.60–1.12)	0.213		
Highest level of education completed				
(≥ high school diploma vs. < high school diploma)	1.01 (0.62–1.65)	0.957		
Employment^a^				
(Yes vs. no)	0.73 (0.56–0.95)	0.018		
Money spent on drugs p/day				
(= $50 per day vs. < $50 per day)	1.53 (1.15–2.02)	0.003		
Drug dealing^a^				
(Yes vs. no)	1.93 (1.50–2.49)	< 0.001	1.76 (1.33–2.31)	< 0.001
Years of using injection heroin at baseline				
(Per year increase)	1.00 (0.98–1.02)	0.781		
Daily non-injection cocaine use^a^				
(Yes vs. no)	1.76 (0.54–5.68)	0.347		
Daily non-injection heroin use^a^				
(Yes vs. no)	7.45 (1.50–37.05)	0.014	8.18 (1.25–53.56)	0.028
Daily crack use^a^				
(Yes vs. no)	1.56 (1.17–2.09)	0.003	1.35 (1.01–1.82)	0.045
Daily meth use^a^				
(Yes vs. no)	1.79 (0.97–3.30)	0.061		
Overdose^a^				
(Yes vs. no)	1.29 (0.77–2.15)	0.328		
Lent syringe^a^				
(Yes vs. no)	0.93 (0.24–3.59)	0.915		
Recent incarceration^a^				
(Yes vs. no)	1.34 (0.83–2.18)	0.233		
Engaged in any form of unprotected sex^a^				
(Yes vs. no)	1.07 (0.64–1.81)	0.787		
Exchanged sex for gifts, food, shelter, clothes, etc.^a^				
(Yes vs. no)	1.30 (0.73–2.32)	0.378		
Attacked, assaulted, or suffered violence^a^				
(Yes vs. no)	1.10 (0.76–1.58)	0.613		
HCV^a^				
(Yes vs. no)	1.05 (0.53–2.05)	0.898		
Plasma HIV-1 RNA viral load > 50 c/mL^a^				
(Yes vs. no)	0.98 (0.76–1.25)	0.853		
On ART (≥ 1 day)^a^				
(Yes vs. no)	0.79 (0.56–1.10)	0.162		
CD4+ cell count^a^				
(Per 100 cells/mL increase)	1.07 (0.99–1.15)	0.096	1.08 (1.00–1.17)	0.042

^a^All behavioural variables refer to the 6 months prior to the follow-up questionnaire

## Discussion

In this study to estimate the prevalence and characteristics of PLHIV who inject drugs that might be eligible for HAT, we observed that approximately one-fifth of all participants were eligible at least once during study follow-up. Periods of HAT eligibility were associated with important factors that have been linked with sub-optimal HIV treatment outcomes in previous studies, including markers of severe SUD, such as high-intensity illicit drug use, homelessness and drug dealing [3, 4, 17]. These associations signal that expanding HAT to this population might influence the factors linked with sub-optimal HIV treatment and improve HIV treatment outcomes, thus contributing to TasP goals [15]. Moreover, they provide further support for the potential role of HAT in decreasing opioid-associated morbidity and mortality.

The links between HAT eligibility and various drug-related (e.g., high-intensity illicit drug use and survival dealing as markers of severe SUD), and socio-structural determinants of health (e.g., homelessness), indicate that HAT-eligible individuals face numerous barriers to optimal HIV treatment outcomes, even in a setting where HIV treatment and care is delivered at no cost [16, 24]. Admittedly, HAT eligibility can impact markers of severe SUD (and indirectly HIV treatment outcomes) but no direct associations with indicators of HIV status were observed. Nevertheless, because a noteworthy proportion of PLHIV met the HAT eligibility criteria in the current study, investment into HAT, and other intensive treatments of this targeted group, might also improve their general medical care outcomes as their commonly comorbid diseases (e.g., HCV) can require intense care and frequent follow-up [1]. Similar impact has been demonstrated through protective effect of methadone maintenance therapy on hepatitis C and HIV incidence among PWIDs in this setting [25, 26]. Another justification for expanding access to HAT among this population lies in the growing body of evidence that confirms that many people who do not respond to typical treatment of opioid use disorder (OUD) improve in HAT in areas such as treatment retention, reduced use of illicit drugs, and social functioning [3]. In response, some settings have started developing guidelines for injectable OAT as a treatment for OUD [27].

We have identified a cohort of PWID with indicators of severe SUD suggesting treatment-refractory opioid use disorders, who could benefit from combined treatment of SUD and concurrent chronic diseases, such as HIV. The association with homelessness, as another potential marker of severe SUD, could suggest that HAT eligible PWIDs cannot afford to stay in housing or access services to facilitate housing [28, 29]. Males in our HIV-positive sample were less likely to be deemed HAT eligible, which is a novel finding, given that men are disproportionately being represented in the opioid epidemic in terms of overdose deaths and in the SUD treatment [30]. In this respect, longitudinal research has identified an adherence gap whereby women living with HIV had lower ART adherence than men which suggest that targeted research into a potential role of HAT in facilitating HAT adherence among women is needed [31]. Studies have shown HIV risk reduction with oral opioid agonist treatment (OAT) [32, 33]. Aligned with the scientific literature, these findings suggest that treatment of SUD should be tailored to the needs of the individuals, including providing supervised injectable HAT to people with treatment-refractory OUD that does not respond to first and second-line oral OAT alternatives [4]. Oral OAT has been shown effective, but not for everyone [34]. For example, between 46 and 65% of patients who initiate methadone OAT discontinue treatment in the first year and relapse to opioid use—a period of high risk for fatal overdose [35]. Furthermore, adherence to therapeutic dose guidelines, which is independently associated with retention, remains problematic in many settings [36]. The benefits of providing concomitant HAT and ART to people with treatment-refractory opioid use disorder, and the potential synergetic effects on treatment outcomes of both diseases, have yet to be established.

This study is limited by several factors. Our sample was not recruited at random and cannot be assumed to represent the larger population of PWID in Vancouver. We did not confirm the diagnoses of mental health and opioid use disorder and did not retrieve provider-level data for treatment episodes. However, the results from previous HAT research did not suggest that a group of PLHIV might not benefit from HAT, because they were not excluded from those studies [2]. Potential risk factors for opioid overdose, such as current co-use of benzodiazepines, alcohol or non-injection heroin, should be explored in future as well. The omission of these factors may have inflated the potentially eligible group. Finally, it is possible that we underestimated the rates of risky behaviours and drug use, such as syringe sharing, due to the effects of social desirability.

To conclude, in our 10-year longitudinal study of PLHIV, approximately 20% of participants would have been eligible for HAT at least once during the study period. Being eligible for HAT was linked to a number of important risk factors for sub-optimal HIV treatment outcomes, and onward viral transmission, suggesting that more evidence is needed for scaling-up access to HAT and how it might contribute to treatment-as-prevention goals.
